# A comparative study of dermatology education in high and low prevalence areas: Kuwait University and the University of Aberdeen

**DOI:** 10.1186/s12909-026-08615-y

**Published:** 2026-01-21

**Authors:** Lulwa AlMulla

**Affiliations:** 1 School of Medicine, University of Aberdeen, Aberdeen, Scotland, UK; 2 Faculty of Medicine, School of Medicine, Kuwait University, Kuwait City, Kuwait

**Keywords:** Dermatology education, Curriculum comparison, University of aberdeen, Kuwait university, Malignant melanoma, Vitiligo, Global health education

## Abstract

**Background:**

Dermatological disease prevalence differs across regions, which may influence how medical schools prioritise dermatology training. Skin cancer predominates in Scotland, while vitiligo is more common in Kuwait. This study compares dermatology curricula at Kuwait University (KU) and the University of Aberdeen (UoA) to examine how local disease prevalence shapes educational focus and student preparedness.

**Objectives:**

To evaluate differences in curriculum content, student confidence, and clinical exposure between KU and UoA, and to assess the global relevance of their dermatology teaching.

**Methods:**

A mixed-methods comparative design was used, combining curriculum document analysis, online surveys of final-year medical students, and semi-structured interviews with faculty and residents. Quantitative data assessed knowledge and confidence, while qualitative themes examined adequacy and exposure.

**Results:**

Both curricula reflected local disease patterns: KU emphasised pigmentary disorders such as vitiligo, while UoA focused on malignant melanoma (MM) and other skin cancers. KU students reported greater confidence with autoimmune and pigmentary conditions, whereas UoA students demonstrated more substantial knowledge of MM and eczema. Both cohorts and faculty identified insufficient dermatology exposure overall.

**Conclusions:**

Dermatology education at KU and UoA aligns with local prevalence but may limit preparedness for managing conditions common elsewhere. Expanding international electives, case-based learning, and global curricular integration may enhance dermatological competency across regions.

What is already known about this topic:

- Dermatology education in medical schools varies widely between countries and institutions.
- Limited exposure to dermatology during undergraduate education can negatively impact students’ confidence and clinical competence.
- There is increasing global recognition of the need to increase dermatology teaching and ensure equitable access to dermatology education worldwide.

What does this study add?

- Provides one of the first direct comparisons of undergraduate dermatology curricula between a UK and a Middle Eastern medical school.
- Demonstrates that differences in curriculum structure, clinical exposure, and assessment methods can influence students’ confidence and career interest in dermatology.
- Suggests practical recommendations for enhancing global dermatology education through improved clinical access and standardized learning outcomes.

## Introduction

Dermatological conditions vary greatly in prevalence across the world, likely influenced by genetic, environmental, and lifestyle factors, including physical activity and dietary habits. For instance, skin cancers like malignant melanoma (MM) are more common in fair-skinned populations such as Scotland, whereas vitiligo is significantly more prevalent in Kuwait, likely due to genetic factors [1, 2]. This variation in prevalence directly affects the emphasis placed on conditions within local medical curricula.

The structure of dermatological education plays a vital role in preparing future doctors on how to diagnose and manage dermatological conditions. A curriculum tailored solely to local disease prevalence risks underpreparing graduates to effectively treat diseases that are more prevalent in other geographical areas. Despite the limited curriculum time, dermatology occupies a central role in everyday clinical practice, studies estimate that 20–40% of general practice visits involve skin complaints [3]. Furthermore, diagnosis of dermatological conditions carries a significant impact which varies widely; delayed diagnosis and treatment of MM carries a high mortality risk [4, 5],while non–life-threatening diseases such as vitiligo can have psychological, social, and quality-of-life impacts [5, 6]. These examples highlight the significance of dermatology competency to ensure both patient and holistic patient-centered care.

Nevertheless, medical students consistently report low confidence in managing dermatological conditions. In one survey, nearly 90% of respondents felt only “slightly confident” or “not confident at all” in dermatology by graduation⁷. This reflects broader structural issues in medical education, as dermatology typically receives few lecture hours and brief clinical exposure. Where placements exist, they are often heavily lecture-based, providing limited opportunities for hands-on experience or real-patient interaction.

Furthermore, educational limitations are compounded by the prevalence-driven distribution of cases. To elaborate further, students are most likely to encounter conditions common in their own country but rarely gain exposure to diseases that are less prevalent locally yet highly significant in other regions. Consequently, it risks students graduating with gaps in knowledge on conditions they may inevitably face in a global setting. This leads to graduates possibly under-recognising severe conditions, delaying referrals, or feeling unprepared to provide care for patients with chronic or psychosocially burdensome skin diseases [7].

A lack of standardisation in undergraduate dermatology education has been widely reported, with major disparities in teaching hours, the balance between lectures and clinical exposure, and the depth of conditions covered⁹. These inconsistencies contribute to inequities in graduate preparedness and raise concerns about whether students receive adequate, globally relevant dermatological training regardless of where they study. In an increasingly interconnected healthcare system, this absence of international standardisation risks leaving gaps in dermatological competence that may impact patient outcomes worldwide.

Although numerous studies have established the epidemiology of dermatological conditions, fewer have examined how medical curricula adapt to these prevalence patterns; even fewer have compared curricula across regions with contrasting disease prevalence. This lack of comparative analysis underscores uncertainty about whether medical graduates are adequately prepared to manage both high- and low-prevalence conditions confidently. Further highlighting the importance of integrating global education to prepare aspiring doctors for practice in diverse international healthcare contexts, rather than limiting their readiness to the local environment of their training institution.

This research paper addresses this gap by evaluating dermatology curricula at Kuwait University (KU) and the University of Aberdeen (UoA) and focusing on conditions with inverse prevalence patterns: skin cancer and vitiligo. Exploring how local disease prevalence influences curricular priorities and student confidence. Through curriculum analysis, student surveys, and faculty interviews, this research provides insight into how prevalence shapes teaching priorities and proposes recommendations to enhance dermatology education and improve student preparedness for global clinical practice.

## Methods

This report was designed using a comparative mixed-methods approach. This report aims to assess how dermatology is taught at both KU and UoA, with a specific focus on low-prevalence conditions within each region, Vitiligo and Skin cancers. A mixed methods approach was chosen because it allows for both quantitative and qualitative data. The former identifies measurable patterns in student self-perceived confidence and knowledge performance, while qualitative interviews provided contextual insight into curriculum structure and perceived preparedness. All quantitative analyses were descriptive and exploratory, given the modest sample size. By combining these methods, the results become more reliable, since information from one source can confirm or add depth to what is found in another.

### Participants

Several groups were included in this study to obtain perspectives across the dermatology education spectrum, including students, graduates, and educators.

Final-year medical students at both KU and UoA were invited to complete an online survey. They were chosen because they have had the greatest exposure to the medical curriculum and are the closest to starting official clinical practice. Although the KU students had not yet begun their dermatology clinical rotation at the start of this study, they had been encouraged to reflect on any prior dermatology experiences in other hospital rotations, such as paediatrics, internal medicine, and infectious disease wards. This approach ensured that both direct and indirect experiences of dermatology could be represented. This difference in training stage was considered during interpretation and limits direct comparability between institutions.

Dermatology lecturers and clinical educators from both institutions were asked to participate in semi-structured interviews. An additional interview was arranged with a dermatology consultant, in particular, who has had both UoA and KU students in his clinic. This was to gain insight into his comparative observations on the differences and similarities between the two groups, particularly in terms of confidence, preparedness, and distinctive strengths.

Additionally, to examine the long-term efficacy of dermatology education, dermatology residents with various medical educational backgrounds were interviewed. These interviews aimed to gain insight into the adequacy of dermatology training, specifically in Kuwait. Questions focused mainly on the benefits and limitations of prior clinical exposure, both within and outside Kuwait, and on shared recommendations for strengthening dermatology education.

Together, the faculty, residents, and students provided complementary insights; the Faculty offered perspectives on curriculum design and intent, while residents and students reflected on preparedness and real-world application. This combination illustrated a comprehensive understanding of the adequacy of dermatology training from undergraduate education through to clinical practice.

### Data collection

Data was collected through three pathways: curriculum analysis, student survey, and interviews.

The Dermatology curriculum was reviewed at both KU and UoA. The analysis focused on lecture hours, clinical sessions, conditions emphasised in learning outcomes, and opportunities for direct patient exposure.

Student Survey – a short online survey was distributed, focusing on both subjective and objective elements. The former was analysed using a series of questions that asked students to rate their self-perceived confidence in managing and diagnosing both Vitiligo and Skin cancers (MM, Basal cell carcinoma (BCC), and Squamous cell carcinoma (SCC)) using a likert scale. The objective element was assessed by evaluating student knowledge using Single Best Answer (SBA) questions, following standard exam formats. Additional questions were included to explore prior exposure to dermatology, and career intentions. Survey items were informed by common undergraduate dermatology learning outcomes and existing medical education literature. SBA questions were designed to reflect standard undergraduate assessment formats and covered core diagnostic principles. Formal psychometric validation and reliability testing were not performed, which is acknowledged as a limitation.

Interviews – Semi-structured interviews were conducted with dermatology educators and residents using a predefined topic guide. Interviews explored perceptions of curriculum adequacy, common student knowledge gaps, teaching methods, and recommendations for improvement.

### Data analysis

Quantitative survey data were analysed using descriptive statistics only. Confidence ratings were summarised using averages and ranges, and SBA performance was reported as proportions correct with denominators stated. No inferential testing was undertaken due to sample size constraints and the exploratory nature of the study.

Qualitative data were analysed using thematic analysis. Transcripts and interview notes were coded by the author, with themes developed iteratively through comparison of responses. Given the small number of interviews, thematic saturation was not achieved, and findings are presented as illustrative rather than representative.

Triangulation across curriculum analysis, survey findings, and interviews was used to enhance credibility.

### Ethical considerations

Formal institutional ethical approval was not required, as confirmed by supervisors at both institutions. Participation was voluntary, no patient data were collected, and all responses were anonymized. The study adhered to the principles of the Declaration of Helsinki.

## Results

### Curriculum analysis

The dermatology curricula differ greatly at both UoA and KU, mainly in the delivery and emphasis of conditions reflecting local health needs. At UoA, dermatology teaching is introduced during the preclinical years, starting with a focused teaching block in Year 2, with around 7–10 days of lectures. Dedicated placements are offered later as an optional placement, in Years 4 and 5. Further exposure to dermatology can be gained during other clinical rotations, such as attending paediatric dermatology clinics. Further dermatological problems are commonly encountered during general practice block. This structure ensures that students encounter skin conditions in various clinical contexts. Learning outcomes mainly emphasise the recognition and management of infectious diseases, such as Scabies and molluscum contagiosum (MC), as well as cancerous skin lesions. Assessments of dermatology conditions are primarily presented in written exams using an SBA format. In contrast, clinical assessments mainly focus on spot diagnoses of skin lesions, particularly MM.

At KU, dermatology teaching also starts during the earlier preclinical years, starting with dermatological manifestations from other conditions, specifically in infectious and sexually transmitted diseases, such as HIV. Focused dermatological teaching is introduced during the later clinical years as a designated week of placement reserved until their final year, as three days dedicated to dermatology clinics. During these sessions, students participate in general and inpatient clinics, multidisciplinary team (MDT) meetings, clinic teaching, and observe or perform practical procedures such as biopsies, patch testing, dermoscopy, potassium hydroxide (KOH) preparation, and cryotherapy. Similar to UoA, additional exposure is gained during other rotations. One day is allocated to paediatric dermatology within the paediatrics block. Assessment is often practical, with Objective Structured Clinical Examination (OSCE) based stations that emphasise the morphology of dermatology. The curriculum particularly emphasises autoimmune and chronic dermatological conditions, which are highly prevalent in Kuwait.

Both universities combine early dermatology teaching during the preclinical years, with dedicated placements towards the end of their medical education, offering opportunities for integrated exposure in other specialities (Table 1). UoA heavily focuses on skin lesions and infectious diseases, while KU emphasises autoimmune and chronic conditions; each institution’s curricula are structured to reflect the local burden of dermatological diseases while also providing students with a broad foundation. KU students are assessed primarily through OSCEs, while UoA students are examined mainly through written questions on lesion recognition and infectious dermatology.

**Table 1:** Curriculum comparison

Asp	KU	UoA
Timing of teaching	Dermatological manifestations (year 1), formal block in final year (year 7)	Year 2, 7–10 days of lectures
Dedicated placement	3 days in the final year (year 7)	Optional 1–2 weeks of placement during the 4th and 5th year as a speciality placement
Integrated exposure	1 day (Paediatric block); MDT meetings	1 day (Paediatric dermatology clinic), GP block
Assessment methods	OSCE-based; spot diagnosis; morphology; investigation/management	SBA; lesion recognition/management
High-Yield conditions	Autoimmune & Chronic (Vitiligo, Psoriasis, etc.)	Skin Lesions ( BCC, SCC, MM), MC, Scabies

### Student survey findings

Thirty-five students were invited to complete the survey: 25 from UoA and 10 from KU. All responses received were included in analysis. As participation was voluntary, response bias is possible, and respondents may not be representative of the wider student cohorts.

**Fig. 1:**
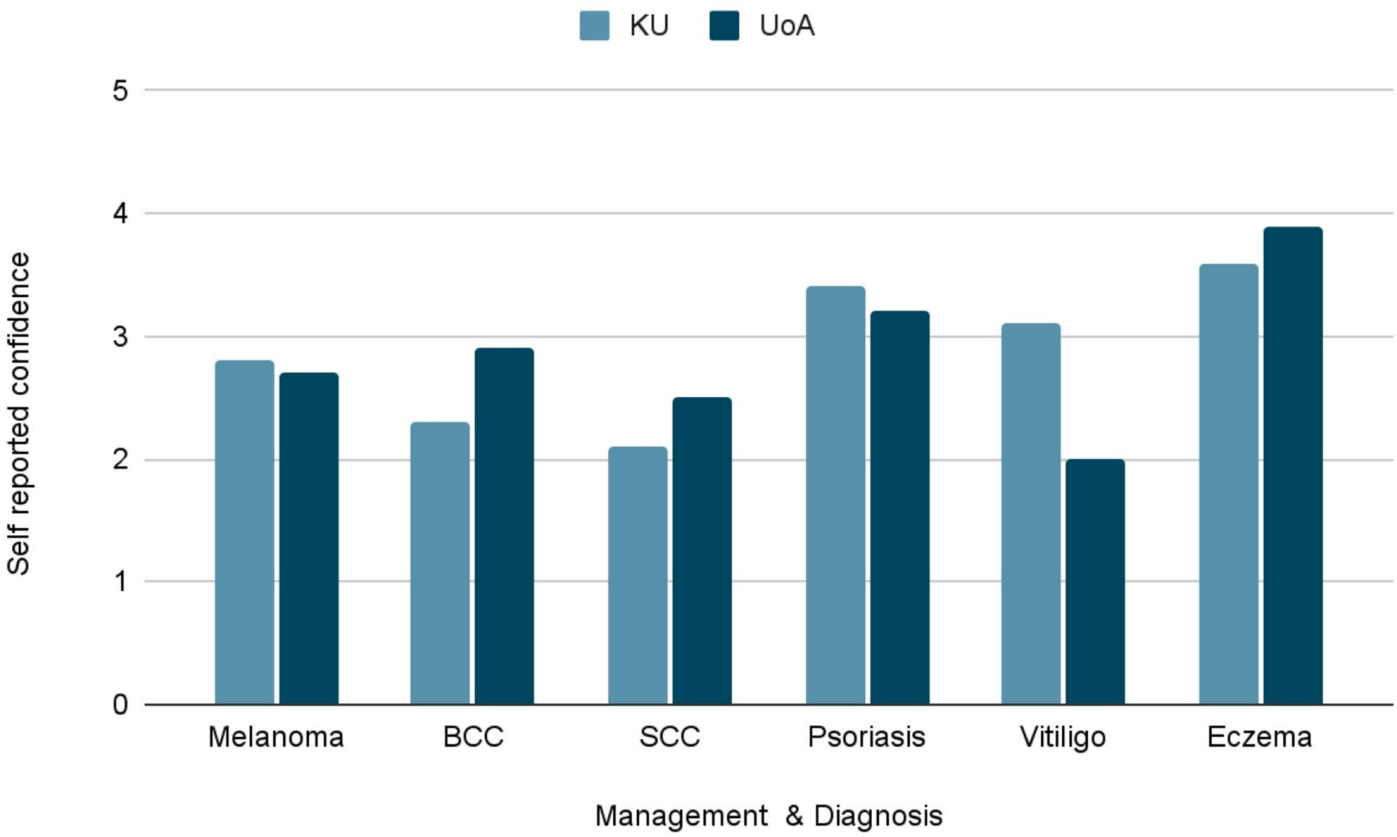
Confidence rating scale - diagnosis & management

Students were asked to fill out to rate their self-perceived confidence in the diagnosis and management of several conditions, from zero to five, with five labelled as “Very confident (fully assured in ability, little to no doubt)”, and zero labelled as “Not confident at all”. KU students reported higher confidence for vitiligo and psoriasis, while UoA students reported higher confidence for basal cell carcinoma, squamous cell carcinoma, and eczema. These findings represent perceived confidence rather than objective competence and are reported descriptively (Fig. 1).

**Fig. 2:**
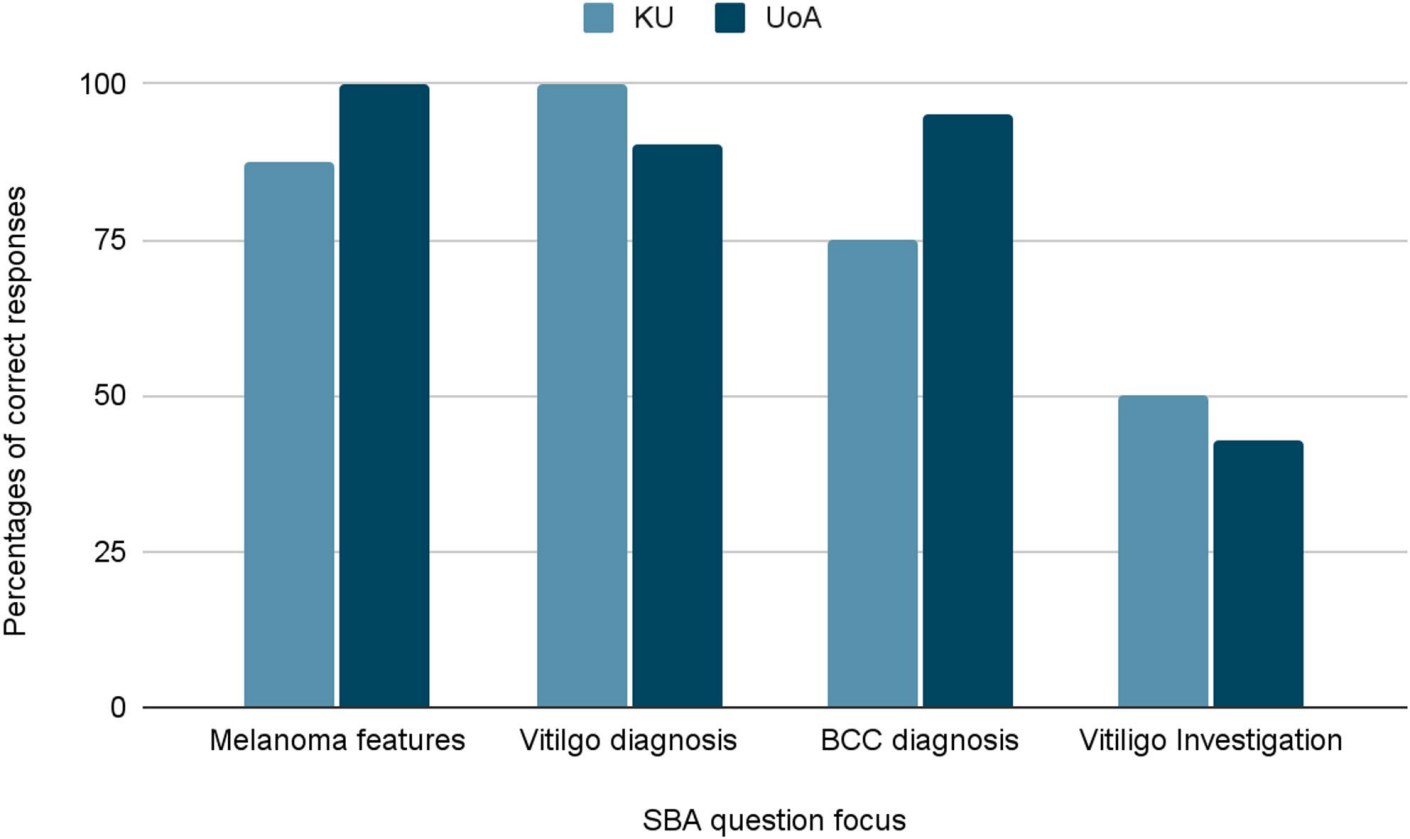
SBA performance across both groups

The responses to the objective data through SBA case-based questions showed a more predictable trend (Fig. 2). 100% of the students from KU correctly answered questions on the diagnosis of vitiligo, compared to 90.4% of the participating students from UoA, which aligns with KU’s higher local exposure. Meanwhile, UoA students achieved stronger results on the skin cancer-related questions, specifically MM, likely due to a stronger emphasis on MM in UoA’s curriculum. Results are presented as proportions correct and should be interpreted as exploratory.

**Fig. 3:**
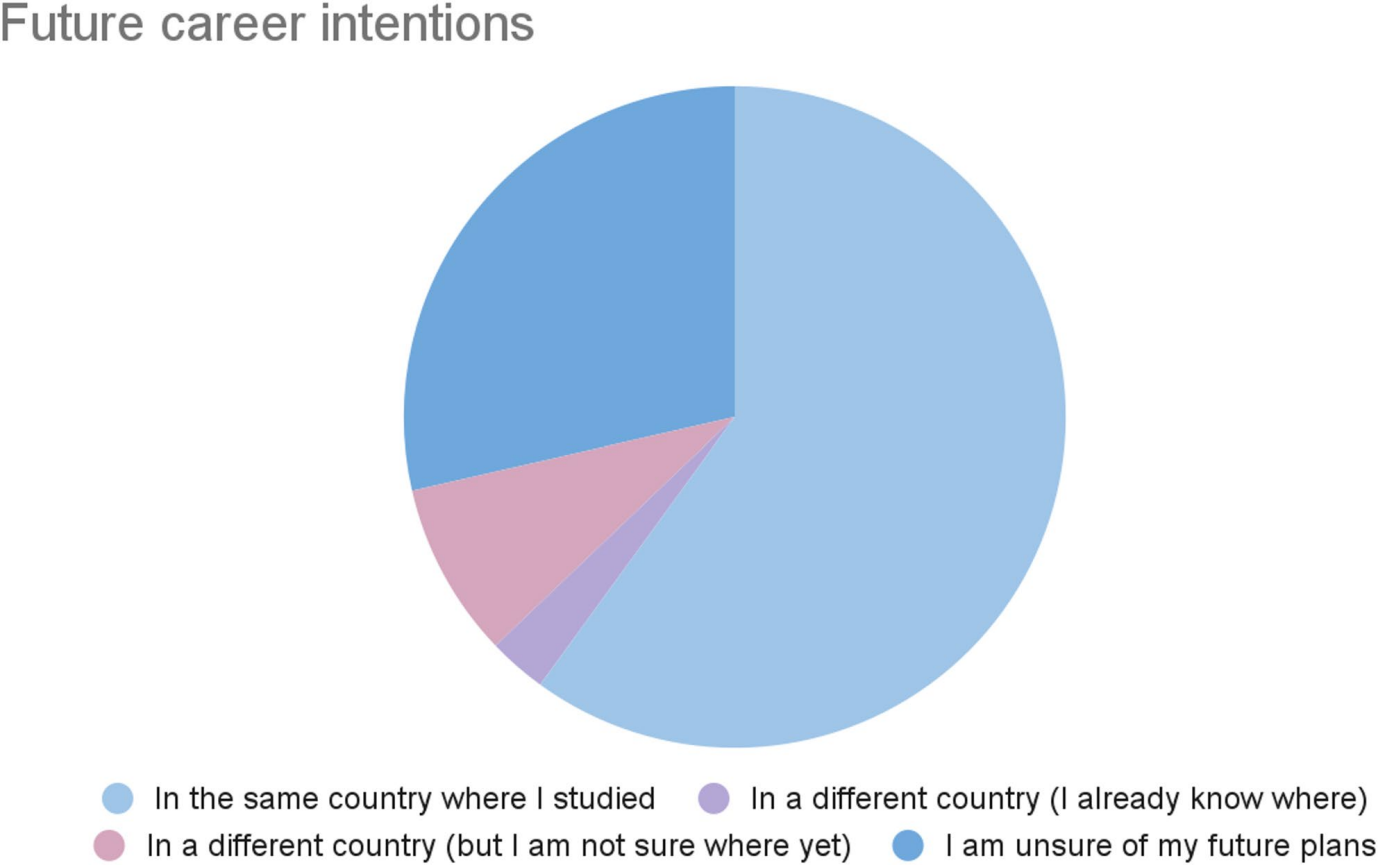
Proportion of students planning to work in the same region they studied, versus those intending to work abroad or undecided

Further responses focused on both clinical exposure and future career intentions. Patterns of clinical exposure varied between institutions. Around 90% of UoA students reported direct exposure to skin lesions, specifically MM, mainly through general practice (GP) and dermatology clinic placements. All respondents had also encountered dermatology through lectures and clinical simulations. However, with vitiligo, UoA students reported brief mentions of the condition in lectures; none reported direct clinical exposure. At KU, on the other hand, 37.5% of respondents reported clinical exposure to vitiligo, with a few noting experience through family members and patients. No students reported clinical exposure to MM, although teaching on the condition had been delivered in lectures throughout earlier preclinical years. These findings suggest UoA students gain more structured exposure to skin cancers, while KU students are more likely to have encountered vitiligo. Both groups, however, identified gaps in balanced exposure across both conditions. When asked about future career plans, approximately 40% of responses stated that they intend to practice in a different location from where they studied (Fig. 3), underscoring the relevance of globally applicable dermatology education.

### Educator and graduate perspectives

#### Resident perspectives

Dermatology residents, who graduated from both KU and the UK, and are currently training in Kuwait, reflected on their undergraduate education, discussing how it has influenced their current training (Table 2). All respondents felt that their undergraduate training provided a strong foundation, although the priority reflected the clinical prevalence of their respective institutions.

**Table 2:** Comparison of themes from resident interviews (UK vs. KU graduates in Kuwait dermatology residency)

Theme	UK graduate	KU graduate (Local)	KU graduate ( with international experience)
Preparedness entering residency	Well prepared, especially in history taking and patient communication.	Well prepared for practice in local conditions; networking.	Solid medical foundation;
Strengths from undergraduate training	Strong exposure to dermatology clinics in the UK; confident in knowledge; high patient load improved communication.	Adequate preparation for vitiligo and other autoimmune skin diseases is common in Kuwait.	Focus on high-yield conditions widely seen in residency.
Knowledge/skill gaps	Needed to adapt to conditions less common in the UK, including pigmentary disorders.	Unclear; undergraduate and residency learning are difficult to separate, as knowledge was reinforced across both stages.	Gaps in dermatopathology, procedural dermatology, underrepresented conditions in local curricula (pigmentary disorders, genodermatoses, and certain infections)
Clinical exposure differences	UK: structured clinics with focus on skin cancers; Kuwait residency: broader case variety.	KU: exposure aligned with local prevalence, especially pigmentary and autoimmune conditions.	Abroad; stronger research integration and subspecialty focus KU; Clinical exposure, high patient volume
Transition challenges	Adjusting to wider spectrum of cases and diverse patient backgrounds in Kuwait.	Smooth transition into residency, with undergraduate foundations strengthened by residency experience.	Needed to adapt to conditions widely seen but not emphasized in teaching
Recommendations for improvement	Cross-regional training valuable.	Highlight multispecialty links (rheumatology, gastroenterology, systemic disease).	Earlier and more structured dermatopathology exposure, integration of procedural dermatology, and broader teaching of less common but globally relevant conditions.

The UK graduate described feeling well-prepared in dermatology-focused history-taking and patient communication, due to structured dermatology clinic placements throughout medical school. They explained that these skills benefited her during her transition into residency. Regardless, they needed to adjust to the broader spectrum of conditions seen in Kuwait, such as mycosis fungoides, which is less commonly encountered in the UK (Resident 2. Dermatology education. [Personal interview, September] Kuwait; 2025 ).

The KU graduate reported that their undergraduate training had prepared them for effective dermatology practice, particularly in clinical skills used in conjunction with knowledge of common conditions seen in Kuwait and in the value of networking (Resident 1. Dermatology education. [Personal interview, September] Kuwait; 2025). While he explained that it is difficult to separate what was learned during undergraduate study from what has since been reinforced in residency, overall, they felt confident in their transition. Further recommendations for aspiring dermatologists and for curricula worldwide include the importance of recognising dermatology in a multispecialty context, noting that skin disease frequently overlaps with other systemic conditions in rheumatology, gastroenterology, and other specialities. Another KU graduate with experience abroad similarly described having a solid medical foundation but feeling “less prepared in dermatopathology, managing rarer or complex diseases requiring multidisciplinary thinking” (Resident 3. Dermatology education. [Personal interview, September] Kuwait; 2025 ).

When reflecting on differences between training locally and abroad, this respondent observed that while KU provided intense clinical exposure and patient volume, international environments placed greater emphasis on evidence-based practice, structured research integration, and adherence to guidelines. He suggested that earlier and more structured exposure to dermatopathology, greater integration of procedural dermatology, and increased teaching of globally relevant but locally rare conditions could strengthen KU’s curriculum and prepare graduates for international competitiveness.

All residents are advised that students interested in dermatology should broaden their knowledge to include globally relevant conditions. They strongly encouraged future trainees to pursue electives in different regions of the world, as this would provide exposure to a broader range of dermatological presentations and complement their local training.

### Mentor perspective

The mentor, a dermatology consultant who has had medical students in his clinic for over 18 years, provided comparative reflections based on his experience with both KU and UoA students. The mentor explained that students from each institution arrive with strong academic foundations. However, as expected, their clinical exposure is shaped by the prevalence of conditions in their respective regions. He noted that, despite UoA students having more general confidence in skin cancer, this does not mean KU students are less capable; instead, they’ve had fewer opportunities to encounter these cases during their training.

In contrast, KU students were more comfortable with pigmentary disorders such as vitiligo, which are highly prevalent in Kuwait. He stressed that this difference should not be viewed as a deficit, but rather as a reflection of geographic variation in disease burden. When asked about improvements, the mentor recommended that both institutions expand opportunities for international electives or fellowships. The mentor suggested that training in various diverse regions to strengthen their global knowledge as future dermatologists (Mentor. Comparing medical students’ education. [Personal interview, September] Kuwait; 2025).

Overall, he concluded that both KU and UoA students are equally well prepared for dermatology practice. While their experiences differ by local prevalence, their adaptability and solid academic foundations enable them to perform effectively in diverse or international clinical settings.

### Faculty insights

Securing multiple faculty insights proved to be challenging at both institutions. This is primarily due to the limited availability of dermatology consultants, whose schedules are heavily constrained by clinical and teaching commitments. As a result, only one response was obtained from each institution. Although these contributions provide essential perspectives on curriculum emphasis and common student difficulties, the individual responses cannot be considered representative of all teaching staff at KU and UoA. Furthermore, given the limited number of interviews, findings should be interpreted as contextual insights rather than representative conclusions.

The educator at UoA described a stronger curriculum emphasis on rashes and skin lesions being the most frequently encountered conditions. Furthermore, students were reported to occasionally encounter vitiligo, mainly in lecture settings rather than in clinics. The most common knowledge gaps seen among students involved diagnosis and long-term management. When asked about teaching methods, the faculty member identified case-based learning (CBL) and online modules as the most effective methods. However, attendance at such sessions was inconsistent, with fewer than half of the students present. They also commented that while additional patient encounters would be valuable, they are difficult to arrange within current teaching structures. No recent revisions or updates have been made to the dermatology curriculum (UoA faculty. Survey on Dermatology Curriculum and Student Learning. [September] Kuwait; 2025 ).

The educator at KU reported that during the dermatology rotation, students attend MDT meetings. They also engage in bedside and clinic teaching where they practice clinical skills, such as cryotherapy. The curriculum emphasises common conditions seen in Kuwait, including papulosquamous and eczematous dermatoses, as well as pigmentary disorders. Exposure to globally significant conditions, such as MM, is limited due to the short rotation. Recent updates have introduced additional conditions to broaden learning, but the educator noted that extending clinical exposure, especially to severe dermatological cases, would enhance knowledge (KU faculty. Dermatology Curriculum and Student Learning. [October] Kuwait; 2025).

## Discussion

This study demonstrates how local disease prevalence and curricular emphasis shape students’ dermatological knowledge and confidence. At UoA, students showed greater self-perceived confidence in identifying skin lesions, particularly MM, reflecting the UK’s higher incidence and curricular focus on cancer detection. KU students demonstrated stronger knowledge of vitiligo and other pigmentary disorders, consistent with their higher local prevalence and curricular prominence.

Interestingly, despite having less clinical exposure, KU students reported higher confidence in managing MM. This indicates that structured, lecture-based teaching can enhance confidence even without direct patient contact. Conversely, UoA students reported lower confidence in managing vitiligo despite lecture coverage, reinforcing that dermatology education is most effective when clinical exposure complements classroom learning [8] .Clinical reinforcement remains essential for sustained competence [9, 10].

These findings carry both local and global implications. Locally, preparedness is influenced by the cases most often encountered during training. Globally, physicians frequently work in diverse healthcare environments where they must manage conditions uncommon in their training region. Thus, curricula focusing narrowly on local prevalence risk underpreparing graduates for international practice.

### Self-reported confidence vs. competence

Confidence does not always equate to competence. KU students reported higher confidence in managing MM than UoA students, yet UoA students performed better on cancer-focused SBAs. KU’s confidence likely stemmed from theoretical familiarity rather than practical experience. Prior research confirms that self-assessed confidence often misaligns with objective competence in dermatology [11, 12]. Practical exposure is a key determinant of diagnostic accuracy, while repeated clinical reinforcement aligns confidence with ability [9]. Addressing this gap requires curricula that integrate case-based learning with sufficient clinical exposure to ensure confidence reflects genuine competence.

### The underrepresentation of dermatology in medical education

Both institutions in this study devoted limited time to dermatology, typically one to two weeks of clinical placements supplemented by exposure during other rotations such as paediatrics or general practice. This reflects a global trend where dermatology remains marginalised despite a high burden of skin disease [13, 14]. Most medical schools allocate fewer than three weeks of dermatology teaching, and some have none at all [8]. In a US survey, only 12% of medical schools had a dedicated preclinical dermatology course, while 36% embedded dermatology within broader teaching blocks [15]. “Curriculum crowding” and limited departmental resources contribute to this restriction9.

Recent reviews call for extended dermatology blocks integrating practical exposure and case-based learning to align with evolving student learning preferences and modern teaching methods [16–18].Yet, dermatology remains underprioritised despite accounting for a significant proportion of primary care consultations [13, 19].Evidence indicates that this underrepresentation directly affects clinical competence [20].Hussain et al.^22^ found that junior doctors reported low confidence in diagnosing skin lesions, reflecting inadequate undergraduate exposure. The current study reinforces these findings, showing that limited teaching and placements risk underpreparing graduates for common clinical conditions.

A KU graduate with international experience further noted limited exposure to dermatopathology and procedural dermatology during undergraduate training. This highlights that short rotations restrict both knowledge breadth and familiarity with subspecialties vital in modern dermatology. Incorporating structured dermatopathology and procedural training earlier in curricula could address this preparedness gap.

### Global education and cross-regional preparedness

The results emphasise the need to view dermatology education through a global lens. Students are most confident in locally prevalent conditions, leaving gaps in readiness for diseases more common elsewhere. All interviewed residents, regardless of background, stressed the value of expanding dermatological knowledge beyond regional boundaries. They advised that students pursue electives abroad to gain broader clinical experience. The resident with international training observed that overseas programmes emphasised evidence-based practice, research integration, and subspecialty training, illustrating how global exposure complements local strengths.

These perspectives align with mentors’ recommendations for electives in Africa and East Asia, exposing trainees to tropical and infectious dermatoses rarely seen in Kuwait or the UK. Structured opportunities for cross-regional training could enhance competence, adaptability, and cultural awareness. Curricula based solely on regional prevalence limit preparedness for increasingly diverse healthcare environments. Global collaborations, shared teaching resources, and inter-institutional electives represent practical strategies to address this gap [14, 21, 22].

### Limitations of the study

Several limitations must be acknowledged. Participation was voluntary, introducing potential selection and non-response bias [23], and sample sizes were modest, limiting generalizability. KU students had not yet begun their dermatology placements, so their responses reflected knowledge from preclinical lectures or exposure in other specialties, possibly affecting accuracy compared with UoA students who had completed formal placements. Faculty participation was also limited to one educator per institution, reflecting the difficulty of involving clinicians balancing clinical and teaching workloads. Qualitative findings were based on a small number of interviews, and thematic saturation was not achieved.

Additionally, this study captures a single point in time and does not reflect how confidence and competence evolve longitudinally. Although resident interviews provided some longer-term context, recall bias remains possible, as it is difficult to separate undergraduate learning from postgraduate reinforcement. Finally, unmeasured factors such as prior electives, online learning, or self-study may have influenced knowledge and confidence levels.

Despite these limitations, triangulation of quantitative and qualitative data provides a nuanced exploratory insight into how dermatology curricula reflect regional disease prevalence.

## Conclusion

This comparative study demonstrates that dermatology education at KU and UoA equips students well for locally prevalent conditions; however, there is variability in exposure to diagnoses that are less common in each setting. Students’ subjective and objective confidence reflected local emphases, Vitiligo at KU and skin lesions at UoA. Notably, KU students’ high self-reported confidence in MM, despite limited clinical encounters, illustrates that structured teaching can bolster perceived readiness. However, their lower objective scores highlight the importance of clinical reinforcement to ensure alignment between confidence and competence. The interview data from faculty, residents, and a mentor converged on the same theme; preparedness is shaped by what students are exposed to, and broader, global training is increasingly necessary as graduates practise in diverse settings.

While findings are context-specific and exploratory, they may cautiously inform curriculum reflection and global dermatology education discussions beyond the two institutions studied, particularly in settings where graduates are expected to practise in diverse clinical environments.

## Data Availability

The datasets generated and/or analysed during the current study are available from the corresponding author on reasonable request. Interview transcripts have been fully anonymised to protect participant confidentiality.

## Abbreviations

KU: Kuwait university
UoA: University of Aberdeen
MM: Malignant melanoma
BCC: Basal cell carcinoma
SCC: Squamous cell carcinoma
MC: Molluscom contagiosum
GP: General practice
SBA: Single-best answer
CBL: Case-based learning
OSCE: Objective structured clinical examination
MDT: Multi-disciplinary team
